# Supplementary figures and images for: Prion infection, transmission, and cytopathology modeled in a low-biohazard human cell line
Source: Life Sci Alliance. 2020 Jun 30;3(8):e202000814. doi: 10.26508/lsa.202000814 (PMC7335386; doi:10.26508/lsa.202000814)

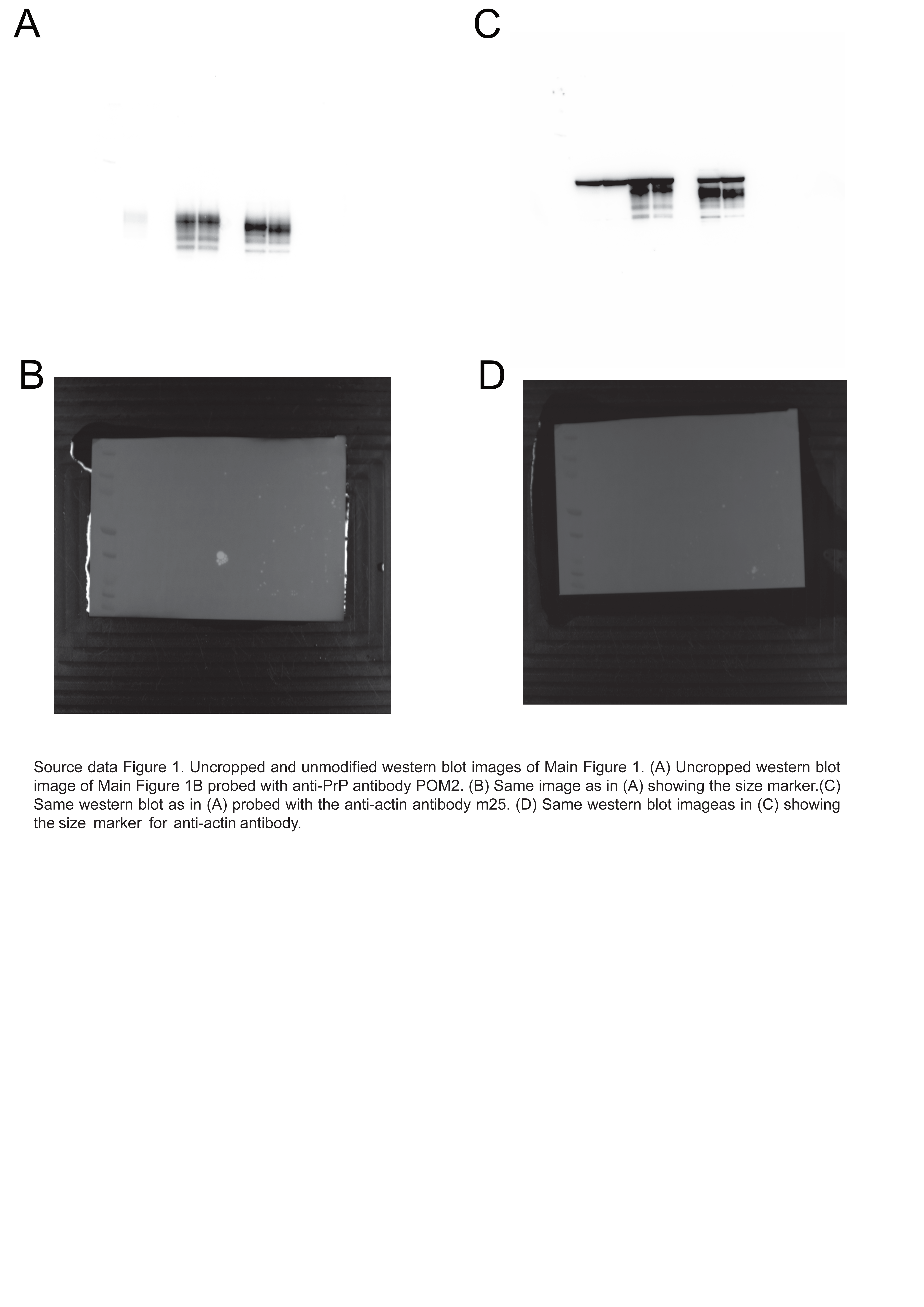

Supplement: Supplementary file 1 [file LSA-2020-00814_Sdata1.tif]

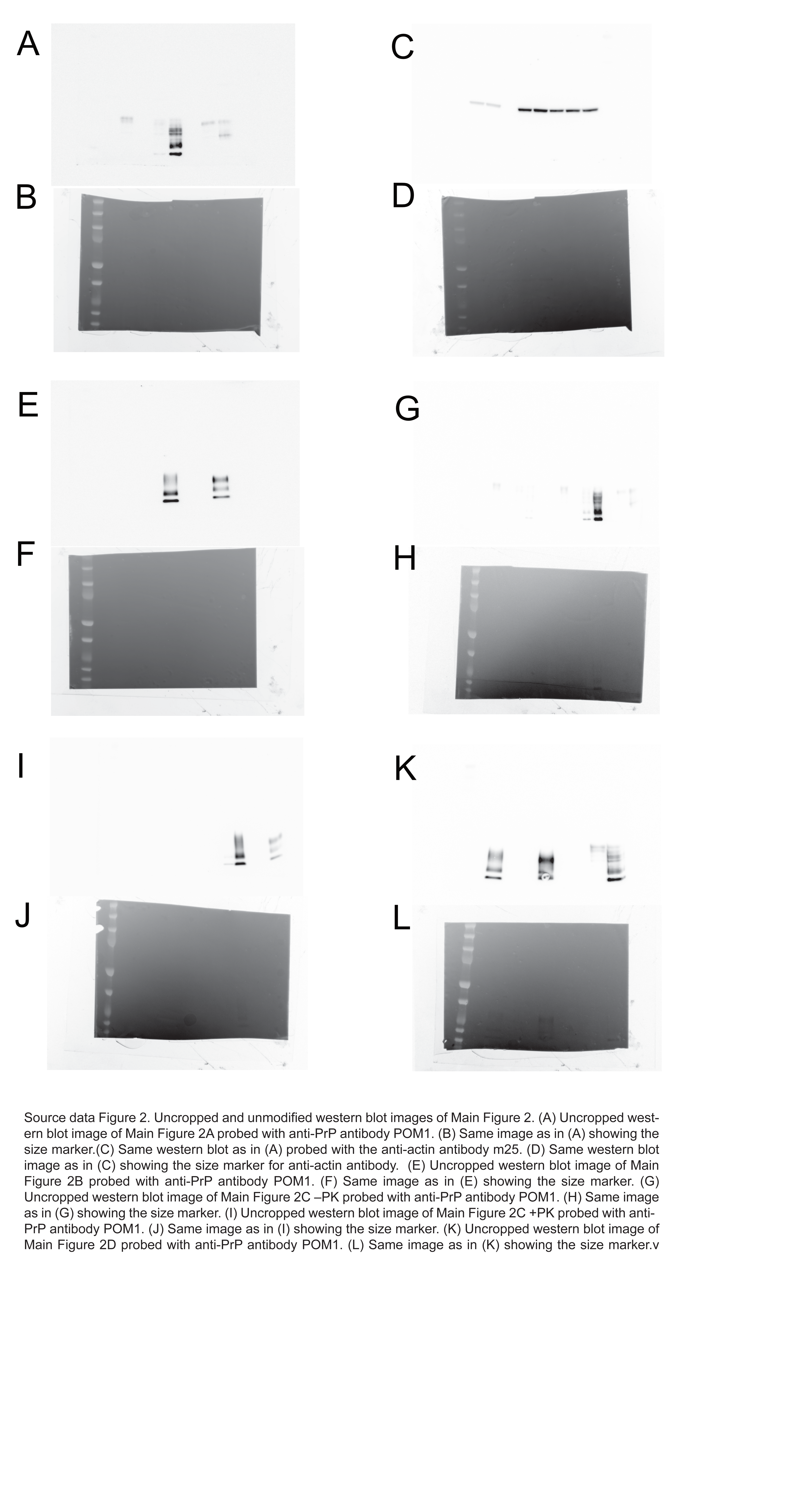

Supplement: Supplementary file 2 [file LSA-2020-00814_Sdata2.tif]

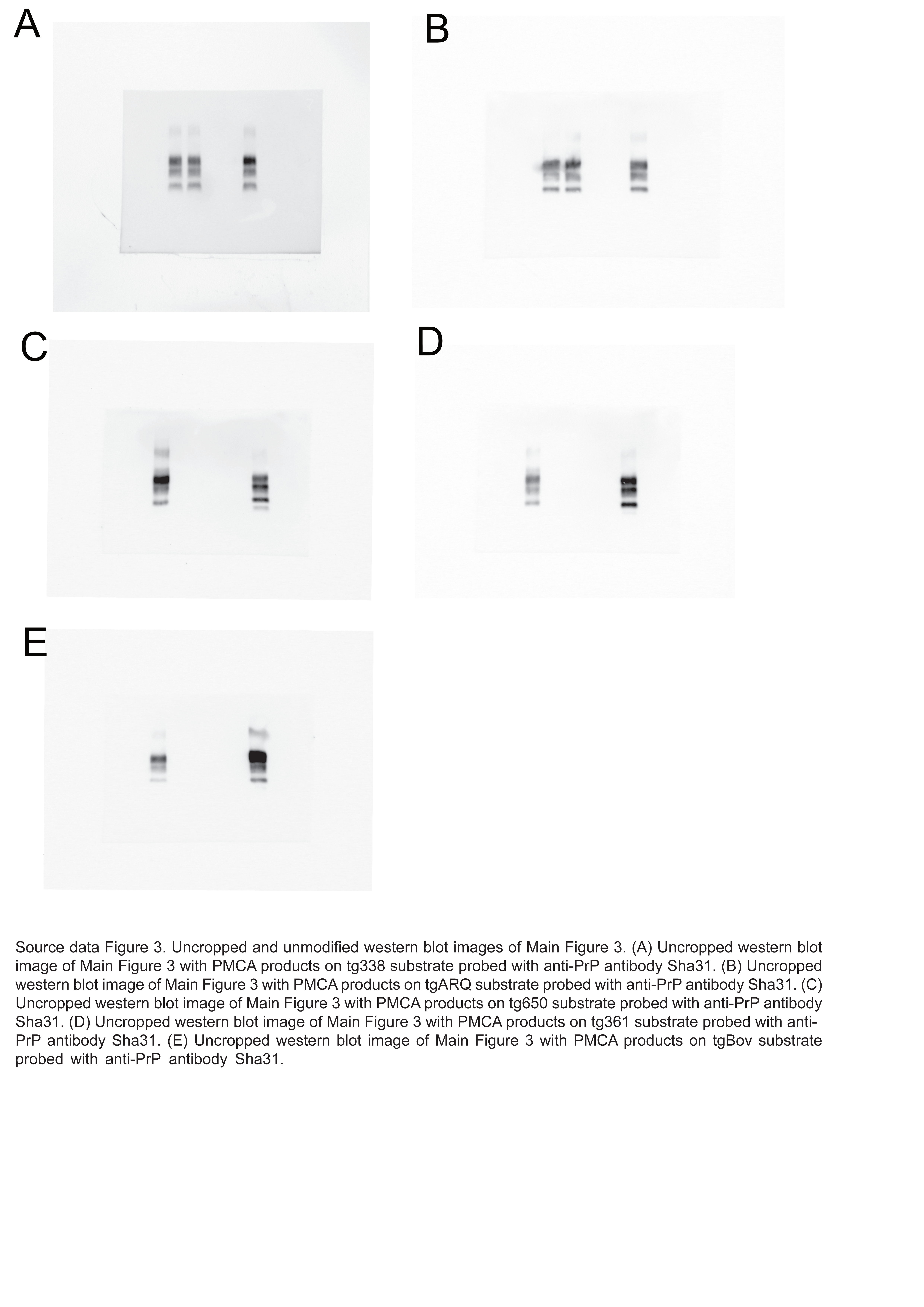

Supplement: Supplementary file 3 [file LSA-2020-00814_Sdata3.tif]
